# Cardiac arrhythmia caused by a novel type of atrial conduction block: A case report

**DOI:** 10.1097/MD.0000000000019264

**Published:** 2020-03-27

**Authors:** Weixun Cai, Siyi Xu, Xiaodong Li

**Affiliations:** a Department Director of ECG; b Department of ECG, Resident physician, Zhejiang Province People's Hospital, People's Hospital of Hangzhou Medical College, Hangzhou, Zhejiang 310014, P. R. China.

**Keywords:** atrial separation, inner atrial block, interference, intermittent atrial dual-directional blockage, P wave block

## Abstract

**Introduction:**

We report an extremely rare case of atrial conduction block with unusual electrocardiogram (ECG) results, which has never been reported before. There are 2 types of atrial conduction block that result in atrial irregularities or complete atrial conduction block. The former is similar to other types of cardiac blocks such as sinus node to atrial block, atrial to ventricular block, or bundle branch blocks, which are characterized by 2 P waves at a specific frequency. This is due to the complete inner atrial block that results in the atrial muscle being divided into 2 parts without conduction between them so that each part has its rhythm generator. The objective of this report is to examine the cause of inner atrial conduction block and to promote awareness of this disorder.

**Patient concerns:**

An 81-year-old Chinese male patient was examined after complaining about chest discomfort, and it was found that he had atrial tachycardia; ECG results revealed a P wave loss at specific intervals (or P wave separation).

**Diagnosis:**

A diagnosis of P wave loss at specific intervals (or P wave separation) was made based on ECG results.

**Interventions:**

An ECG was performed on the patient

**Outcomes:**

It was unclear whether this patient has atrial separation or a new type of atrial conduction block, but our results revealed that this case presents a novel type of atrial conduction block, which we named ‘P wave block.’

**Conclusion:**

The type of EKG shown in this case has never been reported. This EKG shows a new type of conduction block in the atrium, temporarily named as a new type of P wave block.

## 1 Introduction

Cardiac arrhythmia is a common condition of the heart that leads to changes in the electrical impulses that cause an irregular, too fast, or too slow heartbeat that can be life-threatening.^[1,2]^ In the healthy heart, electrical impulses originate from the sinoatrial node and travel to the atrioventricular node (AV node), the bundle of His, right and left bundle branches and Purkinje fibers and reach the ventricular muscle that contracts after electrical stimulation.^[1–4]^ The origin of cardiac arrhythmia can originate at any point of the electrical conduction system in the atrium and ventricle of the heart and may be identified by conducting an electrocardiogram (ECG) analysis.^[5,6]^ Compared to ventricular arrhythmia, the atrial arrhythmia is less well characterized.^[1]^ In our case report, we present a special case of atrial arrhythmia that will help us to understand more about this disease and will help us to develop new treatment regimens for this form of cardiac arrhythmia.

The first wave that is visible in the ECG of a healthy heart is the P-wave that represents the electrical impulses that originate from the sinoatrial node and depolarize the right and left atria.^[7,8]^ The electrical impulses then travel to the AV node where the signal is slowed down while it passes through the AV node, which results in a slower depolarization of the atrium.^[8]^ This short period of electrical inactivity is also referred to as a PR interval on the ECG. The normal range of the PR interval lies between 120 ms to 200 ms measured from the beginning of the P-wave until the beginning of the QRS complex.^[9,10]^

AV node block occurs when the electrical impulses of the heart are significantly delayed in the AV node of the heart or when the signal does not reach the ventricle.^[11]^ It can be classified into 3 different stages: first, second, and third degree heart block.^[12]^ The first degree AV node block is characterized by elongation of the PR interval of 0.2 seconds and higher and a P-wave width of more than 120 ms that rarely results in symptoms in affected individuals.^[13–15]^ The second degree block is a progressive form of AV block characterized by elongation of the PR interval that results in irregular ventricular rhythms and periodic skipping of the QRS complex.^[1,2,16]^ Lastly, the third degree AV block is a complete heart block that results in complete disruption of the electric conductivity between atria and ventricles at the level of the AV node, the bundle of His or in the bundle branches and is characterized by a P-wave width of more than 120 ms and P-waves with 2 opposite phases at lead I, II and aVF.^[3,4,17]^ AV block is diagnosed by analyzing the appearance and duration of the P-wave on the ECG.^[5,6,18,19]^

Due to a complete inner atrial conduction block, the atrial muscle is divided into 2 sections.^[1,3]^ Furthermore, there is a bidirectional conduction block and an activation site in each atrium without any interaction between them. Consequently, the 2 P-waves have a different electric frequency, as determined by ECG. Based on previous studies, it is widely believed that the dual cardiac rhythm is protective as the inbound conduction block is present around the activation site, but the outbound electrical conduction is uninhibited.^[7,8,15,19]^ Therefore, the ECG results show no rhythm change through outside activation which may be modified by this dual rhythm. It is unclear if there is a specific type of atrial conduction block where inbound conduction is intermittently blocked but outbound conduction is completely blocked. In our case report, we describe for the first time atrial conduction block that results in the complete loss of the P-wave. The left atrium is still being activated through reverse conduction coming from the right atrium that leads to a downward P-wave signal at the inferior chest lead.

## 2 Case presentation

An 81-year-old male patient from China with chest discomfort for more than 2 years was examined at Zhejiang Province People's Hospital in Hangzhou, China. The patient had a previous history of hypertension for 19 years and has been taking the drug Nifedipine which stabilizes his blood pressure around 140/90 mm Hg. Ultrasound cardiogram analysis revealed atrial enlargement of 8.6 x 5.8 cm in both sides, normal left ventricular size, and an atrial septum foramen ovale of 0.39 cm. Based on ultrasound cardiogram results, the following diagnosis was made:

(1) Pulmonary valve disease with moderate stenosis
(2) severe tricuspid insufficiency
(3) minimized blood flow from the right to the left atrium (patent foramen ovale), and
(4) mild bicuspid regurgitation.

**Figure 1:**
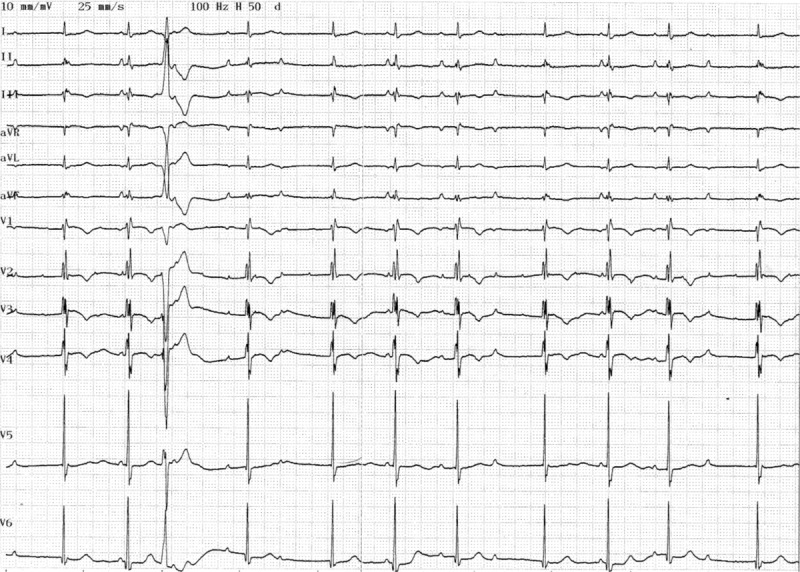
The patient's first ECG recorded with an ECG machine (manufactured in Japan model 9130P). The P wave is upright on I, II, III and aVF leads and is reversed on the aVR lead. The heart rate is 88/min. The distance between P and QRS waves is not regular, in line with the diagnosis of a second degree or higher atrial-ventricular conduction block. On V1, V2 leads, in addition to P wave, there is a small upright wave at a rate of 176/min which is not detectable on other leads. Therefore, we have to rule out that this small wave is caused by an outside interference. ECG = electrocardiogram.

Next, an ECG exam was performed at the ECG lab of Zhejiang Province People's Hospital in China to examine the patient in more detail. To protect the ECG, the room temperature was kept between 12 to 25°C. The patient was asked to lie flat for 10 minutes before the ECG test was performed and was instructed to breathe normally without speaking during the test. The first recording that was measured had a duration of 70 seconds and was performed using the Japanese model 9130. The second recording lasted approximately 30 minutes using a DF-5A ECG machine. The recording speed was 25 mm/s, and the voltage was 1 mV/cm. Written consent was obtained from the patient according to the IRB protocol of the Zhejiang Province People's Hospital. ECG results revealed that the P-wave was upright at lead I, II, III and AVF, as shown in Figure 1. The P wave was reversed with a VR heart rate of 88/min. Based on the distance between the P and QRS wave, a second or third-degree atrial conduction block was suspected. The QRS waves appeared as rsR(s) type at leads V1, V2, and the QRS waves were enlarged close to the end of other leads. The width of the QRS width was 120 ms, indicating a complete right bundle branch block. Wide deformed R3 appeared early without preceding P-waves, indicating premature ventricular contraction. In addition to the regular P-waves, there was a small upright peak at a rate of 176/min at leads V1 and V2. This small upright peak was not seen at other leads. Therefore, the interference factors had to be ruled out first. To confirm the accuracy of these results, a different ECG machine was obtained and used for further examination of the patient. The results of the second ECG showed again a small upright peak at a rate of 176/min, which became more prominent when gain was increased. Some of these peaks appeared in the P-wave section of the low chest leads. We anticipate that this small upright wave is another P-wave due to atrial tachycardia, and it has a different rate from the normal P wave during atrial separation. Further analysis of the patient's ECG results revealed that P- and P’-waves always overlap at the same location. To examine if the 2 P waves are due to atrial separation, the ECG recording time was increased. During the 30 minutes recording time, P- and P’-waves did not change, even though the P-wave frequency altered, indicating that it was not atrial separation that induced the 2 P waves. From these results, we propose that the P’-wave was produced by a different atrial activation site.

Informed consent was obtained from the patient for the purpose of publication.

## 3 Discussion

**Figure 2:**
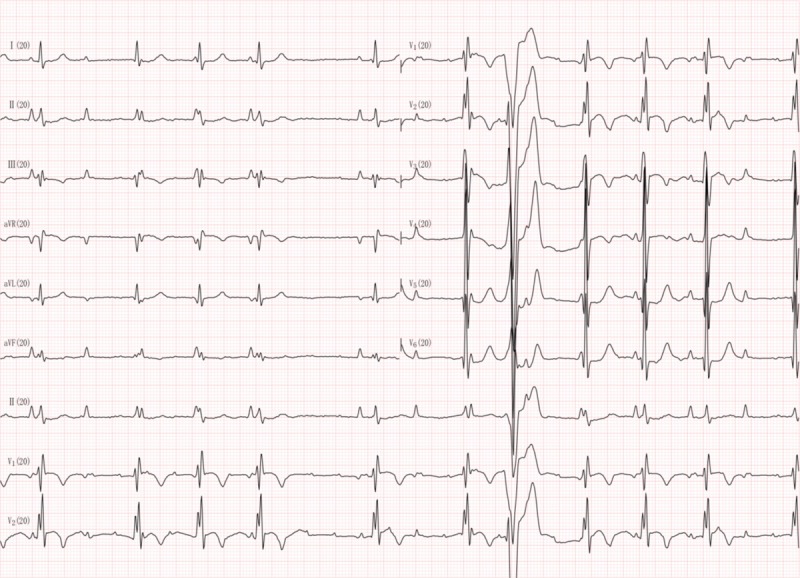
To determine if the small upright wave in Figure 1 1is caused by outside interference, we recorded this patient's EKG again with DF-5A EKG machine with an increased gain to 20 mm/mV. The recording showed the small upright wave again at a rate of 176/mn. This small wave overlaps with an upright P wave on the leads placed in the inferior chest wall. During the 30 min recording period the P1 wave presented consistently with the P wave. The P1 and P wave relationship did not change even when the frequency of P1 was changed. Based on the above observations, we conclude that P1 and P waves are both caused by 1 single ectopic atrial rhythm generator site and is referred to as P’ wave.

**Figure 3:**
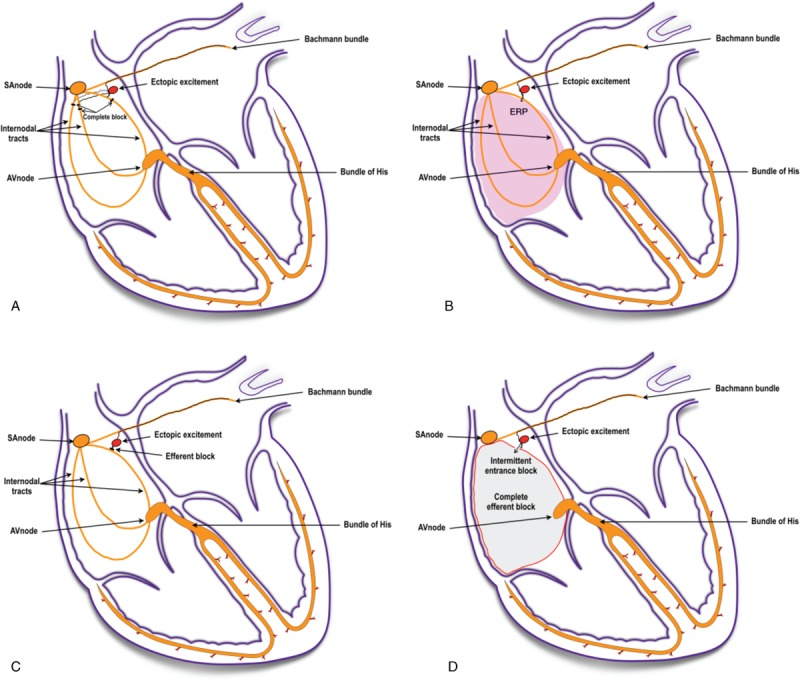
Pathologies of the heart. (A) This graph shows a bundle branch block in the right atrium. (B) Right atrium effective refractory period. (C) The atrial ectopic rhythm generator has 2 exiting sites with intermittent conduction block on 1 exiting site but not on the other exiting site. (D) Intermittent local atrial dual directional conduction block.

In this study, we present the first case of cardiac arrhythmia with 2 P waves, at a rate of 88/min for P1- and 176/min for *P’*-wave. The ECG results of the patient show that the initial P1 wave was upright at lead I, a dip at the V1 lead, and upright at the low chest wall leads. Consequently, we propose that the atrial tachycardia activation site that generates the P*’*-wave is located in the upper section of the right atrium.^[8,20,21]^ While the P1-wave is generated in the right atrium, the P*’*-wave may be generated through depolarization of the left atrium in the presence of a left atrial conduction block, called ‘Bechmann’ branch conduction block,^[2,9,10]^ since in the ECG we observed flat lines at all 12 leads and dual phase at V1 with a duration of P*’* of less than 120 ms. The P1 and P*’*-waves separated at a ratio of 1:2 or 1:4 at the leads after R4, as shown in Figures 1 and 2, which raises the question if the P-wave separation is caused by a second or third-degree conduction block in the right atrium. There are 3 conduction pathways present between the sinus and atrioventricular nodes of the heart.^[11,22]^ If all 3 conduction pathways were intermittently blocked, the P1-wave would disappear due to right atrium depolarization of the heart. However, this scenario is rare. Based on the results of our case study, we believe that such a conduction block is present in the right atrium of our patient (Fig. 3A).

**Figure 4:**
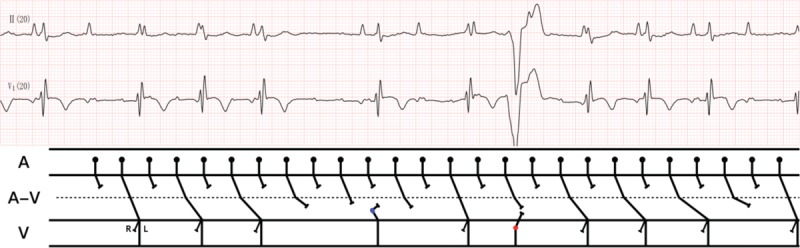
The conduction between the atrium and ventricle as shown in Figure 2.2. According to the trapezoidal graph, P1 and P’ cannot be conducted to the ventricule when they appear simultaneously which indicates that the P1 wave is formed when the signal from atrial tachycardia reaches the atrial location that has an intermittent conduction block. When the ectopic signal reaches the lower part of the atrium or atrioventricular junction, it blocks P’ wave conduction to the ventriculle and causes the P1 and P’ waves of being blocked simultaneously. In addition, the P wave 2:1 conduction block in the upper atrioventricular junction and Wenckebach 4:3 conduction block in the lower atrioventricular junction suggests that there is a P’ wave insidious conduction at the atrioventricular junction.

Furthermore, there is a difference in latency between the left and right atrium of the heart.^[12,14]^ Our patient had severe left and right atrium enlargement as measured by ECG, which may cause latency differences between the 2 atria (Fig. 3B). If latency in the right atrium is longer compared to the left atrium, the ectopic activation at a rate of 176/min cannot activate the right atrium, and this results in the disappearance of the P1-wave and leads to the appearance of the *P’* wave. The atrial ectopic activation site has 2 exits (Fig. 3C) that lead to the transient disappearance of the P1-wave if 1 of the exits is intermittently blocked.^[6,13–15]^ If neither of the 2 exits is blocked, P1 and *P’*-waves may overlap. If the local area within the atrium that is responsible for the P1-wave creation has a complete outbound conduction block (Fig. 3D), then the P1-wave cannot conduct signals to the ventricle to form the QRS wave which can be seen on the ECG (Fig. 4). However, if the local atrial area responsible for P1-wave formation has an intermittent inbound conduction block, then the ectopic stimulation is able to pass through the conduction block, and therefore the P1-wave appears in the ECG. This mechanism is similar to a complete dual-directional block that results in atrial and ventricular separation or is contrary to the protective inbound conduction block of dual cardiac rhythm diseases. The proposed mechanisms outlined in our case study are a possible explanation for the rare occurrence of a ‘P wave block.’ Further physiological studies are needed to verify the mechanisms of P-wave block in patients. The patient refused further examinations due to his advanced age, which make further investigations into the exact mechanisms of this condition difficult. Since atrioventricular conduction blocks do not impact the generation of P-waves, this points to the probability of intermittent atrial dual-directional conduction blockage in our case.

## 4 Conclusions

In summary, P-waves from the same atrial activation site separate into 2 waves, which is different from inner atrial conduction block, dual atrial rhythm, and atrial separation. The case that we present here is clinically extraordinarily rare and has not been given a specific name in the field of cardiology. We propose the name ‘P wave block.’ Based on ECG results, we found the following clinical signs that characterize this condition: atrial tachycardia with P-wave block, layered block at the interface between atrium and ventricle, premature ventricular contraction, and complete right bundle branch block.

## Author contributions

**Conceptualization:** Weixun Cai, XiaoDong Li.

**Data curation:** Weixun Cai, Siyi Xu.

**Formal analysis:** Weixun Cai.

**Investigation:** Weixun Cai, Siyi Xu.

**Writing – original draft:** Weixun Cai, XiaoDong Li.

**Writing – review & editing:** Weixun Cai, XiaoDong Li.

XiaoDong Li orcid: 0000-0001-5056-016X.
